# Manipulation Detection and Preference Alterations in a Choice Blindness Paradigm

**DOI:** 10.1371/journal.pone.0108515

**Published:** 2014-09-23

**Authors:** Fumihiko Taya, Swati Gupta, Ilya Farber, O'Dhaniel A. Mullette-Gillman

**Affiliations:** 1 SINAPSE Institute for Cognitive Science and Neurotechnologies, National University of Singapore, Singapore, Singapore; 2 Institute of High Performance Computing (IHPC), Agency for Science, Technology and Research (A*STAR), Singapore, Singapore; 3 Department of Psychology, National University of Singapore, Singapore, Singapore; 4 Neuroscience and Behavioral Disorders Program, Duke-NUS Graduate Medical School, Singapore, Singapore; National University of Singapore, Singapore

## Abstract

**Objectives:**

It is commonly believed that individuals make choices based upon their preferences and have access to the reasons for their choices. Recent studies in several areas suggest that this is not always the case. In choice blindness paradigms, two-alternative forced-choice in which chosen-options are later replaced by the unselected option, individuals often fail to notice replacement of their chosen option, confabulate explanations for why they chose the unselected option, and even show increased preferences for the unselected-but-replaced options immediately after choice (seconds). Although choice blindness has been replicated across a variety of domains, there are numerous outstanding questions. Firstly, we sought to investigate how individual- or trial-factors modulated detection of the manipulations. Secondly, we examined the nature and temporal duration (minutes vs. days) of the preference alterations induced by these manipulations.

**Methods:**

Participants performed a computerized choice blindness task, selecting the more attractive face between presented pairs of female faces, and providing a typewritten explanation for their choice on half of the trials. Chosen-face cue manipulations were produced on a subset of trials by presenting the unselected face during the choice explanation as if it had been selected. Following all choice trials, participants rated the attractiveness of each face individually, and rated the similarity of each face pair. After approximately two weeks, participants re-rated the attractiveness of each individual face online.

**Results:**

Participants detected manipulations on only a small proportion of trials, with detections by fewer than half of participants. Detection rates increased with the number of prior detections, and detection rates subsequent to first detection were modulated by the choice certainty. We show clear short-term modulation of preferences in both manipulated and non-manipulated explanation trials compared to choice-only trials (with opposite directions of effect). Preferences were altered in the direction that subjects were led to believe they selected.

## Introduction

It is commonly believed that choices are the product of our reasons: we make choices by evaluating available options based on our preferences. As such, our preferences are unaffected by prior choices. However, a number of studies have suggested that the choice process is not so clear-cut. People show increased liking for previously selected options, and decreased liking for previously rejected alternatives [1]–[3] (but see [4]). Such choice-induced preference alterations contradict a major assumption of neo-classical economics: choices reflect preferences, and preferences are unaltered by choices [5].

The phenomenon of ‘choice blindness’ also suggests that our ability to provide accurate explanations for our choices may be much more limited than is commonly believed. Johansson and colleagues (2005) found that, in a two-alternative forced choice task, people can be induced to confabulate an explanation for why they selected the option they did not choose [6], [7]. In their study, participants were shown pictures of pairs of female faces, and asked to choose which face they found more attractive. On a subset of trials the face they selected was re-presented and participants were asked to provide an oral explanation for their choice. On a further subsample of such explanation trials, the faces were covertly exchanged, such that the unselected face was presented as though they had selected it and participants were asked to provide an explanation for choosing it. Only 26% of participants volunteered detection of these manipulations [6]. Similar effects have similarly been shown for selecting the aesthetic beauty of abstract patterns [8], taste of jam/smell of tea [7], moral judgments [9], and political attitudes [10].

There are debates about the extent to which results from the choice blindness paradigm can be generalized to broader questions about introspection and agency [11]–[13]. Johansson and colleagues have made the counter-intuitive suggestion that introspective explanations are confabulatory for both manipulated and actual choices, based on their finding that there are no significant differences in the content of explanations between manipulated and non-manipulated trials [11]. This suggests that in choices such as those explored in choice blindness paradigms, we may lack conscious retrospective access to the reasons for our decisions.

Intriguingly, in Johansson and colleagues (2005 and 2013), they found that preference ratings made immediately after the manipulation led to enhanced ratings for the unselected faces over the selected face [8], [14]. Such an immediate effect of the manipulation on preferences is impressive, but it is unclear to what extent the effect may be an artifact of task structure (i.e., the order and timing of image re-presentation) and whether the preference alteration persists beyond the observed timescale (seconds) to longer durations of minutes or days.

There are numerous questions about what factors drive detection of chosen-face cue manipulations and how they may produce preference alterations. To begin investigation of these questions, we replicated choice blindness in a Singaporean sample, examined how individual and/or trial factors may modulate detection of manipulations, and finally, we examined whether manipulations modulate preferences, and how long lasting such effects may be.

## Methods

### Ethics Statement

All participants provided informed consent under a protocol approved by the National University of Singapore Institutional Review Board.

### Participants

The experiment consisted of two phases. Thirty-two participants (16 females; age range = 19–30 years, age mean±SD = 22.2±2.4 years; self-identified ethnic background = 29 Chinese, 2 Indian, and 1 Vietnamese) took part at Day 1, and received S$10 for their participation. For Day 2, participants were re-contacted and invited to perform a follow-up online survey, for a chance to win a lottery of S$40. Twenty-five participants (13 females; age range = 19–26 years, age mean±SD = 21.9±1.7 years; self-identified ethnic background = 23 Chinese, 1 Indian, and 1 Vietnamese) completed Day 2 through the online survey approximately two weeks after their participation at Day 1. Participants were collected across two sampling periods, resulting in two participants receiving S$40 prizes.

### Experimental design

During Day 1, participants completed 1) a choice blindness task, 2) an attractiveness rating task, 3) a similarity rating task, and 4) behavioral surveys. Computerized behavioral tasks (1–3), were presented using Matlab (Mathworks, Inc.) and the Psychophysics Toolbox [15]–[17]. For Day 2, participants performed an online attractiveness re-rating via SurveyMonkey (http://www.surveymonkey.com/).

**Figure 1:**
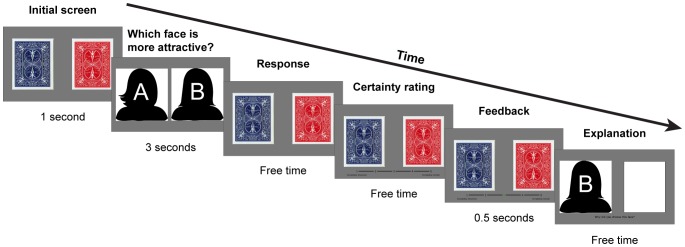
Choice blindness task. On each trial, participants choose which of two faces they found more attractive. Each trial began with presentation of the backside of two playing cards presented on the left and right sides of the screen. These two cards were flipped, replaced by a pair of faces for three seconds. Then, the playing card backsides were re-presented, and participants reported which face they found more attractive by pressing a corresponding key (free response time). Of note, while the figure utilizes cartoon face silhouettes (Pixabay, Inc.), the actual task utilized color photographs of human faces (see *Methods*). Immediately after their choice, they rated their choice certainty by keyboard response, with a brief green highlight indicating the selected certainly level. On 10 out of 20 trials, participants only made their choice and rated their certainty (Choice-only condition; C trials). On the other half of the trials, they were asked to provide textual explanation for their choice after the certainty rating. On 5 of the trials, the face they had selected was shown (Non-manipulated condition; NM trials). On the other 5 trials, they were presented with the non-selected face as though they had selected it (Manipulated condition; M trials). Note, as requested, the specific faces shown were not the ones utilized in the study (these are from the Aberdeen face set (www.pics.stir.ac.uk).

In each trial of the choice blindness task, participants select the more attractive of two presented faces (Figure 1). Twenty pairs of color photographs of Caucasian female faces were presented, with randomization of the pair presentation order across subjects. Face images were derived from the LUCS face database (Lund University Cognitive Science), courtesy of Dr. Petter Johansson. Of note, due to copyright issues the illustration of the task in Figure 1 utilizes cartoon face silhouettes (from Pixabay, Inc).

**Figure 2:**
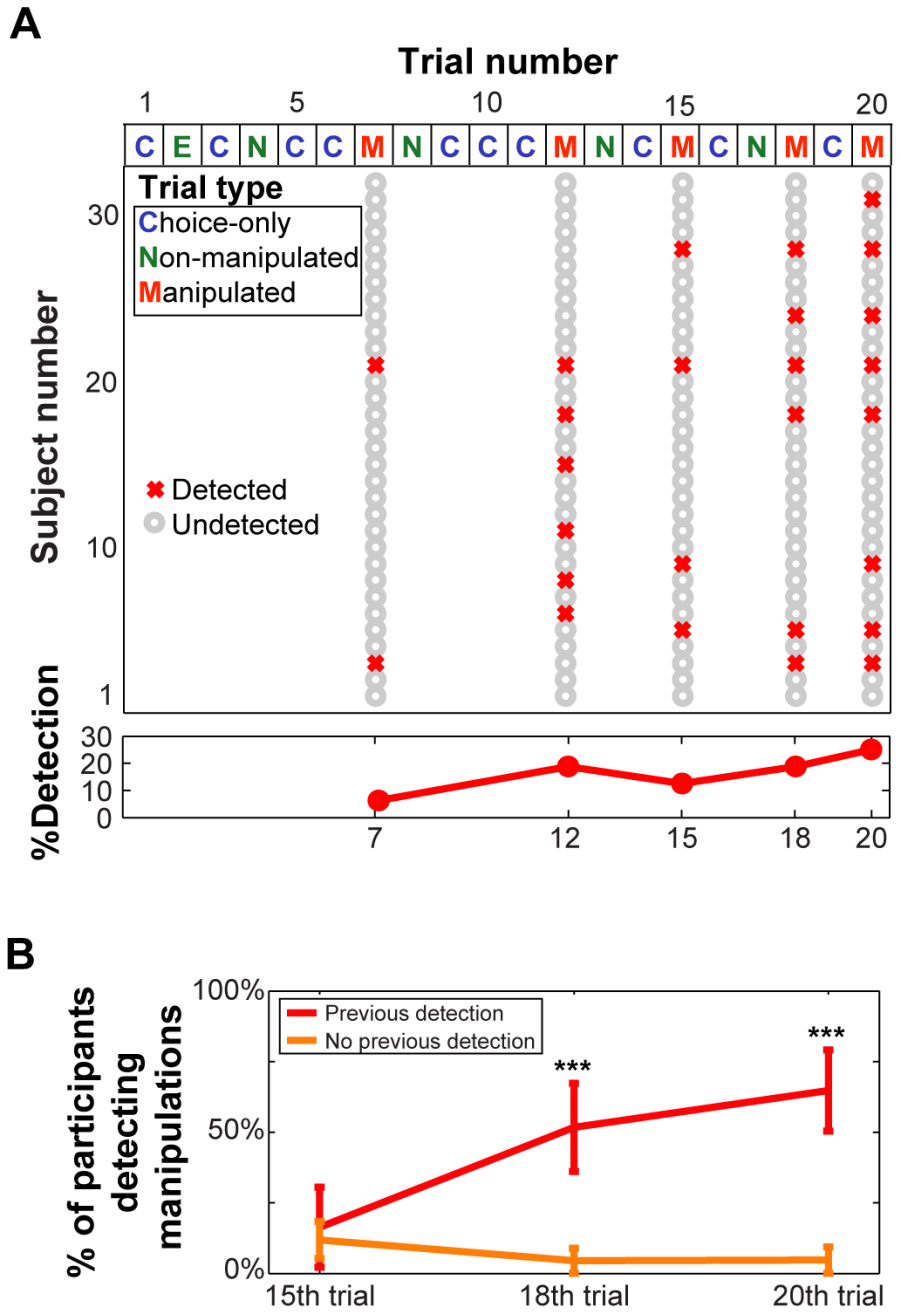
Manipulation detection. A) Task trial structure depicting fixed order of Choice-only (C), Non-manipulated (NM), and Manipulated (M) trials (on top). Detection of chosen-face cue manipulations on M trials is depicted, with grey Os indicating non-detections of manipulations and red Xs indicating concurrent detections of manipulations. At the bottom, we have depicted the proportion of participants who detected cue manipulations in each trial. B) The proportion of participants detecting cue manipulations separately for previous detection (PD) and non-previous detection (NPD). Significant differences between these groups at each trial were determined using permutation tests, and significant differences are indicated (***, p<0.001).

Each trial began with the visual presentation of the backsides of two playing cards (1 second), followed by a presentation of a pair of female face images (3 seconds). The backsides of the cards were re-presented, and participants reported which face they had found more attractive (keyboard press, free response time). Immediately following their response, they were asked to rate their certainty about their choice on a scale of 1 (“completely uncertain”) to 5 (“completely certain”) (keyboard press, free response time). Ten of the twenty trials ended at this point (Choice-only condition; C trials). On the remaining 10 trials, following the certainty rating, participants were asked to type an explanation of why they found their selected face more attractive. On 5 of these 10 explanation trials, the chosen face was re-presented throughout the explanation phase (Non-manipulated condition; NM trials). On the other 5 trials, the non-selected face was presented as though it were the face the participant had selected (Manipulated condition; M trials). Trials on which participants provided textual explanations, and those on which their choices were exchanged were presented in fixed order (Figure 2a, top), with the order of presented face pairs randomized across participants.

Following the choice blindness task, participants engaged in two additional computerized tasks. In the attractiveness rating task, each face was presented individually and participants rated the attractiveness of each face on a scale of 1 (“very unattractive”) to 9 (“very attractive”) (keyboard press, free response time). The selected rate was highlighted with green color for 0.5 sec. Faces were presented in random order, without information about which pair they had been presented in or the choice the participant had previously made. Within the instructions, participants were presented with a few example faces of individuals of higher and lower attractiveness than the faces used in the task, to anchor the extremes of the attractiveness scale.

In the similarity rating task, on each trial, a pair of faces was presented in random order, and participants were asked to rate the similarity of the two faces on a scale of 1 (“very different”) to 9 (“very similar”) (keyboard press, free response time). Task instructions included examples of highly similar and highly different face pairs (outside of the attractiveness range of the face stimuli used in the task) to anchor the extremes of the similarity scale.

After completing the tasks, participants completed pen and paper documents one-page-at-a-time, with the first requesting simple demographic information. The second page started with the question “If you have any comments about what could be improved in the tasks that you just did, please provide them here:”, with several lines of space for the participant. The second question was answered yes/no, “Did you notice any problems with the task?”, and then “If you answered yes, please describe the problems as clearly as possible here:” with several lines of space, and then one more question, “How often did they occur?” Participants then completed two surveys, the Cognitive Reflection Task (CRT) [18], and the Need for Cognition/Faith in Intuition (NFC/FI) [19]. The CRT assesses an individual's ability to suppress an intuitive, spontaneous response, while NFC/FI is a self-assessment questionnaire for measuring an individual's reliance on effortful cognition (NFC) and intuitive decision making (FI). We hypothesized that individuals who are more inclined to rely on intuitive judgments might be less likely to detect the manipulations, since they would be less likely to have explicitly rehearsed reasons for their choice which would conflict with the manipulated choice.

The final pen and paper page began by describing to participants the overall manipulation that took place during the choice blindness task, but described it as though there were two groups: “If you were in Group 1, the face that reappeared on the screen after you made your choice was always the one selected as the more attractive. If you were in Group 2, on a few trials the face that reappeared was NOT the one you originally selected as the more attractive.” Participants were then asked to state which group they thought they had been in. In fact, all participants were in Group 2. Following the question, participants that indicated they believed they were in Group 2 were asked to provide a count and describe in writing any instances in which they noticed the manipulation ‘for sure’ and, separately, any instances in which ‘something seemed odd’. Participants were then fully debriefed, and explicitly asked whether they would like for their data to be removed from the study (No participants opted for their data to be discarded). Finally, participants were asked if they had previously heard about the experiment (none had), and were asked not to discuss the experiment with other possible participants.

For Day 2, participants were invited by email to perform an attractiveness re-rating task online (Surveymonkey, Inc.). 25 participated, taking the survey 9–29 days after their Day 1 participation (mean interval±SD = 16.1±4.4). The 40 faces from Day 1 were re-presented in random order for participants to re-rate their attractiveness on the same scale, with the same initial instructions.

The attractiveness ratings (within day), choice certainty ratings, and face similarity ratings were z-scored within subjects for further analysis. In order to examine preference alterations, we calculated the attractiveness difference by determining the difference between z-scored attractiveness ratings for the selected and unselected faces for each trial within each day. MATLAB software (The MathWorks, Inc., Natick, MA) was used for all analyses.

## Results

### Manipulation detection

Our first question was whether choice blindness could be replicated within a Singaporean sample using a computerized choice blindness task. To this end, we examined whether participants indicated awareness of chosen-face cue manipulations. Following the methods of prior studies, we examined whether participants volunteered awareness of the manipulation immediately after the manipulation (concurrent detection), by examining their typewritten explanations for statements for evidence of detection (such as ‘I chose the other face’ or ‘I didn't choose this face’). We also categorized one additional trial as concurrently detected where the participant verbally reported detection.

Concurrent detection occurred on 16.9% (*N* = 27) of the 160 manipulated trials performed across all participants (5 trials×32 participants). This detection rate is comparable to the 13% observed in Johansson's original work [6]. At least one trial of concurrent detection was indicated in 37.5% of participants (*N* = 12). Of note, the false-positive rate for concurrent detection was zero, with no explanations on non-manipulated trials suggestive of problems with the stimuli.

Our post-task query about general problems yielded an additional 37.5% “retrospective detection”, i.e. participants (*N* = 12) voluntarily reporting manipulations as some sort of problem, bringing the total up to 75.0% detection (*N* = 24). When told that there were two groups, in one of which the unselected face was presented as the cue on a few trials, 81.3% of participants (*N* = 26) exhibited “cued detection”, classifying themselves into the cue-manipulation group. While these figures may be useful as an upper bound on detection rates, we cannot be sure how many of these retrospective and cued “detections” were in fact guesses or confabulations, and so in our analysis we focus on the concurrent detections.

When participants concurrently detected a manipulation, they often still failed to report subsequent manipulations (58.3%, *N* = 7 of 12). In fact, only one participant concurrently reported all five manipulations.

### Predicting detection across manipulated trials

To begin investigation of manipulation detection across trials, we plotted detection of manipulations across all trials for each participant (Figure 2A), including the proportion of participants concurrently detecting at each manipulated trial. Overall, the proportion of participants reporting manipulations increased as the task progressed.

We next examined which trial factors predict detection of manipulations across all manipulated trials. We concatenated manipulated trials across participants, and performed a stepwise regression of manipulation detection by factors of choice certainty, attractiveness difference, face similarity, manipulation trial number (1 to 5), and the number of prior detections. The final model included two of these factors (*p*<0.0001, *F* = 131.9, Adjusted *R^2^* = 0.62), the number of prior manipulation detections (*coeff* = 0.36, *p*<0.0001, *t* = 15.97), and the manipulation trial number (*coeff* = −0.027, *p* = 0.048, *t* = −2.02,). Detection increased strongly with prior detections, but also decreased weakly with exposure to more manipulated trials.

To visualize how first detection increased the tendency to detect future manipulations, we calculated the proportion of participants that detected the manipulation on each trial separately for those that had previously detected manipulations and those that had not (Figure 2B). We omitted data for the first manipulation trial as only two participants detected this manipulation. To estimate the standard error of each sample, we performed a bootstrap analysis (10,000 random re-samplings of 26 of the 32 participants, without replacement). The proportion of participants detecting manipulations was higher for those with previous detections on every manipulated trial, significantly so for trials 18 and 20 (permutation tests, all combinations tested, *p*s<0.001) [20].

### Predicting detection after first detection

As the number of prior detections had such a large effect on future detections, we investigated how trial factors influence manipulation detection following first detection. Using stepwise multivariable linear regression analysis of concurrent detection on each trial, with factors of choice certainty, attractiveness difference, and face similarity across detected and undetected trials following first detection, in participants with concurrent detections (*N* = 12). We excluded data from one participant who only detected the last manipulated trial. All manipulation trials subsequent to the first detection were included in this analysis, resulting in 14 detected trials and 16 undetected trials. The final model (*p* = 0.041, *F* = 4.60, Adjusted *R^2^* = 0.11) found participant's choice certainty rating to be a significant predictor of detection following first detection (*coeff* = 0.21, *p* = 0.04, *t* = 2.15) while their attractiveness rating (*coeff* = 0.01, *p* = 0.22, *t* = 1.27) and similarity rating (*coeff* = −0.02, *p* = 0.83, *t* = −0.22) were not significant predictors.

### Individual determinants of concurrent detection

We examined which individual traits might account for individual variability in manipulation detection through multivariable linear regression analysis on rates of concurrent detection with gender of the participants, and the scores of CRT, NFC and FI as independent variables. Examined across all manipulation trials, stepwise regression revealed none of these factors significantly explained individual variability of concurrent detection rates. Further, a chi-squared test for independence revealed no significant relationship between individual concurrent detection (detector vs. non-detector) and gender (*p* = 0.47, *Χ* ^2^_1_ = 0.53), indicating no gender difference in the number of concurrent detectors and non-detectors.

We also examined whether individual factors could account for how many manipulated trials participants encountered before their first concurrent detection. This stepwise regression was limited to participants with at least one detection; testing individual factors of gender and survey scores from CRT, NFC, and FI. None of the factors were able to predict the number of manipulations that participants concurrently detected.

### Preference alterations

**Figure 3:**
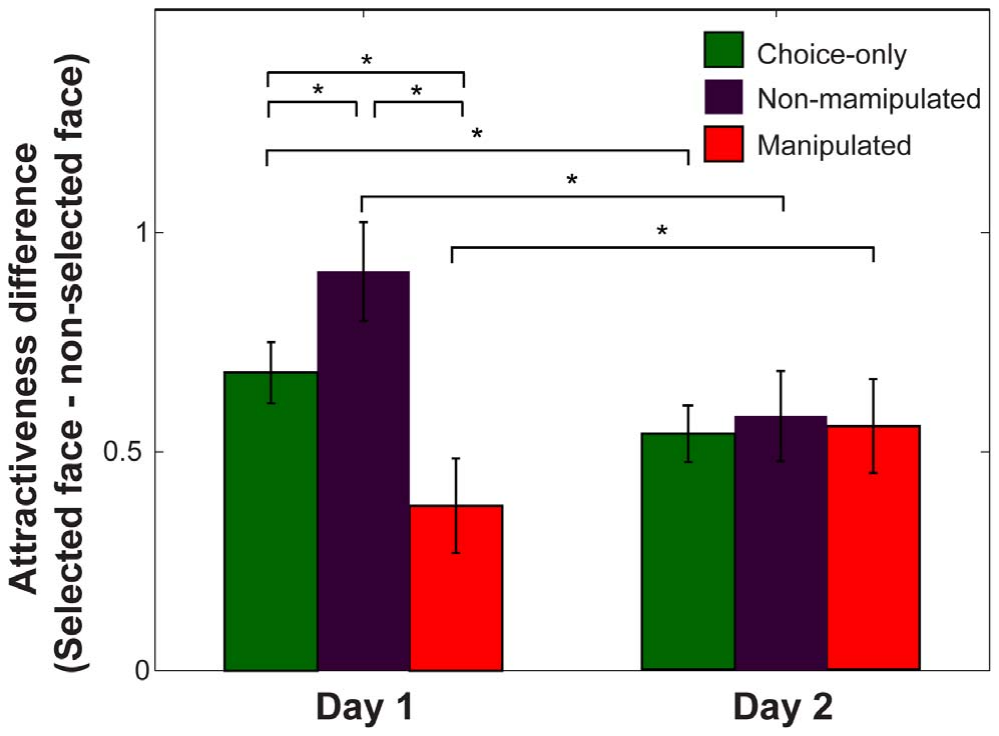
Preference alteration. Differences of rated attractiveness between selected and unselected faces for each trial type (C, NM and M trials) at each day (Day 1 and Day 2). Significant differences were found between all comparisons of trial types on day 1 (NM>M, M<C, and NM>C), with no differences between trial types on day 2, and comparisons between days for all trial types (C and NM: Day 1>Day 2, M: Day 1<Day 2).

To examine the effects of chosen-face cue manipulations on preferences, and modulation of these effects over Day 1 and Day 2, we compared the attractiveness difference between selected and unselected faces on C, NM, and M trials at each day (Figure 3), with a 3×2 repeated measures ANOVA with trial type (C, NM, M) and Day (Day 1, Day 2) as the independent variables. This analysis consisted of 25 participants who completed the online attractiveness re-rating at Day 2. The normalized attractiveness rating differences (Mean±SE) were 0.68±0.07 on C, 0.91±0.11 on NM, and 0.38±0.11 on M trials at Day 1, and 0.54±0.06 on C, 0.58±0.10 on NM, and 0.55±0.11 on M trials at Day 2. We found a significant interaction between trial types and day (*p* = 0.001, *F_2,48_* = 7.61) with no significant main effects (trial type: *p* = 0.07, *F_2,48_* = 2.88; Day: *p* = 0.12, *F_1,48_* = 2.59). Post-hoc tests showed a main effect of trial types at Day 1 (*p* = 0.003, *F_2,48_* = 6.63) with no significant effect at Day 2 (*p* = 0.93, *F_2,48_* = 0.07). A Tukey's HSD test on the attractiveness difference at Day 1 revealed significant differences among all combinations of trial types (*p* = 0.05; NM>M, M<C, NM>C). Taking C trials as baseline, this pattern indicates enhanced preference for actually chosen faces in non-manipulated trials (NM>C) and decreased preference for the actually selected face in manipulated trials (M<C). In other words, participants showed enhanced preference for the face they were led to believe they selected. Also, the attractiveness differences were significantly different across the two days, within each trial type, as revealed by paired t-tests (C: Day 1>Day 2, *p* = 0.03, *t_24_* = 2.29, NM: Day 1>Day 2, *p* = 0.02, *t_24_* = 2.47, M: Day 1<Day 2, *p* = 0.04, *t_24_* = −2.16). At Day 2, both C and NM trials showed decreased differences while M trials showed enhanced differences. To ensure that the comparison of C trials between Day 1 and Day 2 was unaffected by our normalization of the rated attractiveness within day, we re-normalized the attractiveness difference by combining across days within each subject, and confirmed the significant difference for C trials was maintained (Day 1>Day 2, *p* = 0.007, *t_24_* = 2.96).

Detection of the chosen-face cue manipulation could potentially alter subsequent attractiveness ratings. We tested this possibility by comparing the attractiveness difference between detected (*N* = 26, mean±SE = 0.52±0.23) and undetected trials (*N* = 34, mean±SE = 0.22±0.17) from the twelve participants that concurrently detected cue manipulations, revealing no significant differences (t-test, *p* = 0.28, *t_58_* = 1.09).

To test whether manipulations lead to individuals preferring their unselected face to their selected face, we examined the rate of preference inversions across trial types. Inversions were defined as instances in which the participant chose one face, but then rated the unselected face as the more attractive of the two in the subsequent attractiveness rating task. Across all trials without manipulation (C and NM trials), participants inverted their preferences on an average of 14.6% of face pairs. On manipulated trials, this increased to an average of 23.8%. Although this increase was large on average (61.3%), a bootstrap analysis (10,000 iterations, resampling with replacement) revealed no significant difference between these preference inversion rates (*p* = 0.20).

A potentially significant confound is that the time each face is presented could enhance familiarity, driving enhanced preference for the re-presented face (the mere-exposure effect). We tested this possibility by correlating the duration of exposure to each face stimuli during the choice explanation phase with the attractiveness difference between selected and unselected faces, showing no significant correlation (*r* = 0.006, *p* = 0.92).

The median temporal separation between face choices and attractiveness ratings was 369.4 sec (±160.5), calculated as the average difference between the middle of each of the two tasks across participants.

## Discussion

We replicated the phenomenon of choice blindness within a Singaporean sample utilizing a computerized task. We show that the majority of participants failed to concurrently detect chosen-face cue manipulations. Further, we examined what factors predict manipulation detection, showing that detection increased following first detection, and that subsequent detections were predicted by the rated choice certainty for each trial. We also found alteration of preferences on explanation trials (manipulated and non-manipulated) in the direction participants were led to believe they chose. This modulation of preference, while present in the short term (minutes), did not sustain in the long term (days or weeks).

### Replicating Choice Blindness

Though not intended as a pure replication, this study uses a core task structure closely resembling that of the original Johansson *et al.* choice blindness experiment [6]. Notable differences include 1) computerized task presentation; 2) typed (instead of oral) choice explanation; 3) more M trials per participant; 4) a Singaporean sample engaging in cross-ethnic rating (predominantly Southeast Asians rating Caucasian faces). Participants exhibited choice blindness under these conditions, with a concurrent detection rate (16.9%) consistent with that observed in the original study (13%). To our knowledge, this is the first replication of choice blindness in a predominantly non-Caucasian sample.

### Predicting manipulation detection

We investigated what factors influenced detection of chosen-face cue manipulations, examining both individual differences and trial factors for possible effects. No individual factors (gender, CRT, NFC, and FI) were able to predict individual manipulation detection (detection rates, or number of manipulated trials before first detection). Examining trial factors, prior detection of cue manipulations strongly increased the likelihood of additional manipulation detections (Fig. 2b). Such an increase suggests that participants may have become more vigilant following first detection (cascading detection effect) [6].We also found a weak effect in the opposite direction, indicating that as participants performed more manipulated trials they became less likely to notice the manipulation.

While on average the rated attractiveness difference was greater on detected than non-detected trials, it was not a significant predictor of detection subsequent to first detection (*coeff* = 0.01, *p* = 0.22, *t* = 1.27). This suggests that the immediate feeling of decision engagement (certainty) for the trial is a better predictor of manipulation detection than the delayed (∼6 minutes) attractiveness ratings.

Most participants who reported a manipulation failed to detect some subsequent manipulations. We examined whether task factors (choice certainty, attractiveness difference, and face similarity) influence subsequent detection, finding a linear relationship between choice certainty and the likelihood of detection. The modulation of subsequent detection by choice certainty could potentially be due to enhanced memory for the presented faces as a result of higher decision engagement. If so, detection could be the result of the comparison of the re-presented face with the face the subject remembered selecting (or rejecting). Alternatively, certainty could reflect awareness or memory of the subject's decision process. This could promote manipulation detections via recall of specific feature differences that played an explicit role in that process (i.e. the subject could notice an anomaly at the level of focal features rather than at the level of the final choice). Further, detection could potentially be the result of idiosyncratic feature preferences that render the chosen-face cue implausible to the subject, without requiring memory. For example, a subject might have an established distaste for a specific feature of a presented face (hair color, jewelry, etc.), and not believe that they would have selected that face regardless of what other option was presented.

Failure to detect manipulations does not mean that participants have no memory of their choice processes, nor that they were unaware of the reasons for their choices. For example, Petitmengin and colleagues recently suggested that participants have memories for their choice process, although introspective access requires mental effort [21], [22].

While it may appear strange that some of the participants failed to detect subsequent manipulations after first detection, choice blindness is not the only paradigm in which subjects often fail to notice alterations of stimuli. In change blindness, an inspiration for early studies of choice blindness [8], people often fail to notice a change in a visual stimulus even when forewarned that such a change will occur [23]–[25]. While change blindness could be the result of failures of low-level perception or attention [26], choice blindness suggests this unawareness extends to higher cognitive functions.

### Preference alterations

We examined whether chosen-face cue manipulations result in alterations of preferences, and the temporal duration of any such effects. We found that preferences, as measured by the difference in later attractiveness ratings between selected and unselected faces, were altered for both manipulated and non-manipulated explanation trials, compared to choice only trials (Figure 3). The preference for the actually selected face was increased for NM trials (NM>C), and decreased for M trials (M<C), showing enhanced preference for the faces participants were led to believe they selected.

As our preference alterations were found using an attractiveness difference measure (selected minus unselected), this could be the result of either increased attractiveness for the faces that participants were led to believe they selected and/or decreased attractiveness for the other faces. Our task design is unable to differentiate between these possibilities.

While preference alterations were found in the two studies by Johansson and colleagues [8], [14], it is possible that these were produced by a shared confound in the study design. Immediately after their choice or explanation subjects were shown each face again individually and asked to rate its attractiveness, but crucially, the “chosen” face (actual or manipulated) was always presented first. Thus, simple satisficing heuristics to save cognitive effort could potentially both produce failures to detect manipulations and result in preference inversions (rating the unchosen-manipulated face higher than their actually-chosen face). For example, in Johansson and colleagues (2008), participants could simply rate the first face more highly than the second face. Although Johansson and colleagues (2013) found the attractiveness difference was higher for NM than M trials at a second rating, which was conducted with randomized order after the second choice task [14], the first rating, made immediately after explanation, could still influence the second rating. The present study avoids these issues by moving all attractiveness ratings to a separate task, following all choice trials, with no structural cues to the options selected or the face pairings. Such a difference in experimental design could have resulted in a lack of preference alterations within our study, if the effects were solely due to the order artifact. Interestingly, while we did find preference alterations, compared to the rate found in the study by Johansson and colleagues [8], we not only saw a lower rate of preference inversions, but also found no significant difference in the rate of inversions between M and NM trials.

There are multiple possible, potentially non-conflicting, cognitive explanations for our found preference alterations. Firstly, subjects' beliefs about their choices (choice beliefs) may directly modulate preferences leading to increased preference for the option a participant believes they chose [27]. In non-manipulated trials the chosen-face cue may reinforce their belief in their actual choice, driving the greater difference found in NM trials over C trials. In manipulated trials, the chosen-face cue manipulation may replace their remembered choice, resulting in the decreased difference found in M trials over C trials. Intriguingly, if choice beliefs are forgotten over time, this model would also suggest that the C trials would have enhanced difference values on Day 1 over Day 2, which we also found. Of course, a major assumption of this model is that participants have information about their choices and modulate these choice beliefs based upon the presented chosen-face cue information. The strength of, or participants' access to, such memories is called into question by participants' failure to detect manipulations.

Secondly, preferences may be modulated by the focused engagement with the presented face that occurs during the process of providing a choice explanation. Whether veridical or confabulatory, choice explanation may engage a variety of cognitive and behavioral processes, potentially including memory retrieval, rationale construction, and motivated scrutiny of the face itself [28], [29]. The observed preference modulations could thus result from subjects spending time attending selectively to the positive aspects of the presented face and constructing or recalling reasons for preferring that face. (While Johansson and colleagues found preference alterations in a paradigm with no explanation phase [8], as noted above the effect observed in that case may be due to order of presentation.)

Thirdly, cognitive dissonance theory would suggest that preferences will be altered in such a way as to reduce any cognitive dissonance produced by necessity of providing explanations for choices inconsistent with their preferences [1], [2], [30].

Finally, the preference alterations were all in the direction of the number of presentations of each stimulus, raising the possibility that they are the result of increased familiarity with the presented face, which may enhance preference for the presented face (the mere-exposure effect). However, we found no relation between the duration of additional exposure to each face during the chosen-face cue and variation in the attractiveness difference. Further, studies demonstrating the mere-exposure effect for novel stimuli, as in classical conditioning, normally require several repetitions of presentation [31]. Note, in the Johansson study of preference reversals [8], all faces were presented an equal number of times, further suggesting that the familiarity effect cannot be the sole cause of our found preference alterations.

Recent studies have shown that such preference modulation can also occur without choice or even without a ‘sense of agency’ [12]. For example, in the ‘endowment effect’ simple ownership enhances the value of a good: people randomly given an object set a higher price when invited to sell it than those not given the object will offer when invited to purchase it [31]. Further studies are needed to clarify whether a common mechanism is involved across such preference alterations.

We found no differences in attractiveness ratings between trial types at two weeks after the choice task. This is suggestive of an extinguishing of alterations within two weeks. This result is both surprising and expected, given recent studies. Surprising, given a recent study by Sharot and colleagues which suggested that choice-induced preference changes could sustain for over two years [32]. Expected, as other studies have shown that choice-induced preference changes can be unstable, and can even be removed by simply washing one's hands [32].

## Conclusions

We successfully replicated the phenomenon of choice blindness in a Southeast Asian sample (Singaporean) using a computerized choice blindness task. Detection of chosen-face cue manipulations was strongly influenced by the number of prior detections. However, even after first detection, many participants failed to detect manipulations on subsequent trials. Subsequent detection was predicted by choice certainty, potentially related to decision engagement. We show clear short-term modulation of preferences in both manipulated and non-manipulated explanation trials compared to choice-only trials. Manipulated and non-manipulated trials had opposite-signed modulations such that preferences were altered in the direction that subjects were led to believe they selected.
